# Response Surface Methodology for Optimization of Buspirone Hydrochloride-Loaded In Situ Gel for Pediatric Anxiety

**DOI:** 10.3390/gels8070395

**Published:** 2022-06-22

**Authors:** Marwa H. Abdallah, Dina M. Abdelnabi, Hanaa A. Elghamry

**Affiliations:** 1 Department of Pharmaceutics, College of Pharmacy, University of Ha’il, Hail 81442, Saudi Arabia; 2 Department of Pharmaceutics and Industrial Pharmacy, Faculty of Pharmacy, Zagazig University, Zagazig 44519, Egypt; dinaabdelnabi@zu.edu.eg (D.M.A.); haelghamry@zu.edu.eg (H.A.E.)

**Keywords:** in situ gelling, buspirone hydrochloride, sodium alginate, HPMC-K15M, factorial experimental design

## Abstract

The purpose of the current investigation was to formulate, assess, and optimize oral in situ gels of buspirone hydrochloride (BH) with the specific end goal of expanding the time the medication spends in the stomach, thereby ensuring an extended medication discharge. This would allow the use of a once-a-day dose of liquid BH formulations, which is ideal for the treatment of pediatric anxiety. In situ gels loaded with BH were prepared using various concentrations of sodium alginate (Na alg.), calcium chloride (CaCl_2_), and hydroxypropyl methylcellulose (HPMC K15M). The in situ gels exhibited the desired consistency, drug distribution, pH, ability to form gel, and prolonged drug release in vitro. The (3^3^) full factorial design was utilized for the revealing of the ideal figures for the selected independent variables, Na alg. (X_1_), HPMC (X_2_), and CaCl_2_ (X_3_) based on measurements of the viscosity (Y_1_) and percentage drug release after 6 h (Y_2_). A pharmacokinetic study of the optimum formulation on rabbits was also performed. The formulation containing 2% of Na alg., 0.9% of HPMC-K15M, and 0.1125% of CaCl_2_ was selected as the ideal formulation, which gave the theoretical values of 269.2 cP and 44.9% for viscosity and percentage of drug released after 6 h, respectively. The pharmacokinetic study showed that the selected oral Na alg. in situ gel formulation displayed a prolonged release effect compared to BH solution and the marketed tablet (Buspar^®^), which was confirmed by the low C_max_ and high T_max_ values. The optimum oral Na alg. in situ gel showed a 1.5-fold increment in bioavailability compared with the drug solution.

## 1. Introduction

Buspirone hydrochloride (BH) is an anxiolytic drug and an example of drugs that are easily absorbed from the GIT. It has a short half-life (2 to 4 h) due to first-pass metabolism [1] bringing about a quick exit from the blood flow; therefore, various dosages are required. To avoid this problem, oral extended-release formulations are typically utilized, as these release the drug slowly into the gastrointestinal tract (GIT) [2]. A comparison between a controlled-release and an immediate-release formulation of BH showed an almost 3.3-fold higher plasma concentration at a steady state following the extended-release dose and a relative bioavailability of 170–190% compared to a similar dose of an immediate-release formulation [3]. This was explained by the fact that BH is mainly metabolized by first-pass metabolism in the gut wall. Since BH is released from the immediate-release formulation at a much faster rate than from the extended-release formulation, more BH is metabolized [4]. This explains the need to develop an extended-release oral dosage form of BH.

Oral liquid dosage forms are ordinarily viewed as the most favored forms of drug administration [5]. The optimization of oral drug delivery systems for young patients is a major challenge. The majority of pediatric patients aged 6 to 11 years are unable to swallow solid oral dosage forms. As a result, finding an easily swallowable dosage form for children is critical [6]. Oral in situ gel is an innovative mucoadhesive drug delivery system that takes the form of low-viscosity liquid upon formulation but transforms into gel under certain conditions in the body (pH, temperature, etc) [7]. As a result, it not only extends the contact period between the medication and the absorptive sites in the stomach, but also allows the drug to be released slowly and continuously, making it particularly effective for chronically used treatments [8]. Drug delivery systems that are formed in situ are simple to manufacture and easily swallowed, especially by children [9].

Alginate polymers contain β-D-mannuronic acid and α-L-glucuronic acid residues linked by 1,4-glycosidic linkages. The interaction between glucuronic acid in alginate chains and Ca^2+^ ions causes the gelation of alginate solutions [7].

Experimental design is a form of design in which some variables can be evaluated at several levels in a definite number of investigations. Experimental designs are divided into two types: factorial design and response surface design. Factorial design is a form of screening design, which classified into full factorial design and fractional factorial design; response surface design includes central composite design and Box Behnken design [10]. Box Behnken design (BBD) is an operative software related to response surface methodology (RSM), which is based on designing experiments and studying models via mathematical and statistical equations in addition to specific graphical forms [11].

This study attempts to discuss the formulation and optimization of oral in situ gels for the sustained delivery of BH in order to achieve a reduced daily dose frequency. Gastro-retentive in situ gelling liquids were formulated using different concentrations of Na alg. and HPMC. Calcium chloride is the most commonly used chemical agent to bind alginate molecules together in the preparation of in situ alginate hydrogels from alginate solutions in water [12]. Sodium citrate is added to form a complex with free Ca^2+^ in the formulation to maintain its fluidity until it reaches the stomach, where Ca^2+^ starts to leach from the formulation in response to the acidic environment, causing Na alg. to shape into gel [13].

In the present study, a sustained oral delivery system of sodium alginate in situ gel for buspirone HCl was developed. Its viscosity, drug distribution, pH, ability to form gel, in vitro and in vivo animal study were explored.

## 2. Results and Discussion

### 2.1. DSC Studies

**Figure 1:**
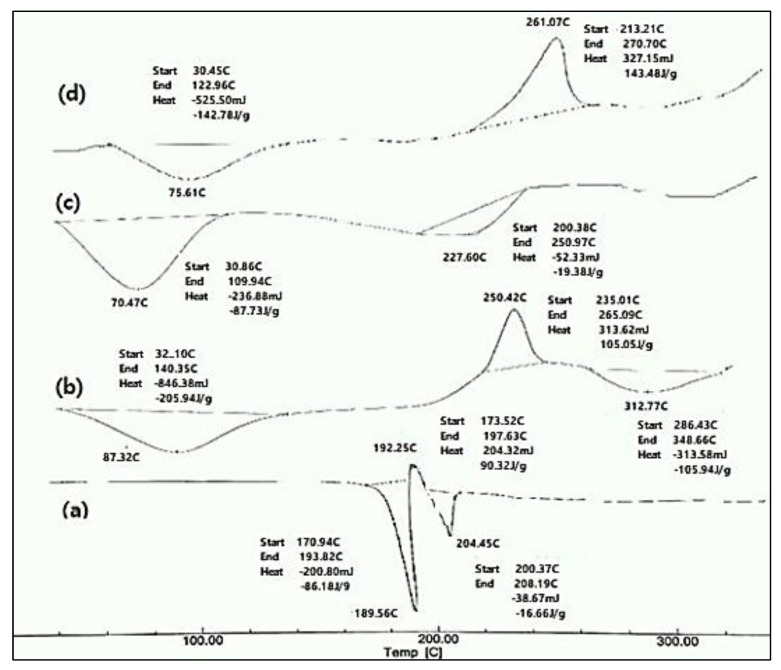
DSC thermograms of (**a**) pure drug (BH), (**b**) Na alginate, (**c**) HPMC K15M, and (**d**) physical mixture of the drug with Na alginate and HPMC K15M.

The DSC charts of the pure BH, pure Na alg., and pure HPMC K15M, as well as their physical mixture, are shown in Figure 1. The DSC thermogram of the pure drug showed two endothermic peaks at 189.56 °C and 204.45 °C, and one exothermic peak at 192.25 °C. The endothermic peak at 189.56 °C was due to the melting of the pure drug sample. The exothermic peak at 192.25 °C was due to the conversion of the polymorphic form-1 of the drug to polymorphic form2. The second endothermic peak, at 204.45 °C, was due to the melting of the recrystallized polymorph-2 of the drug. These results were in accordance with the data obtained in the literature [14,15]. The thermogram of the Na alg. showed a broad endothermic peak at 87.32 °C, which may have appeared due to the loss of the water content and moisture of the polysaccharide, and an exothermic peak at 250.42 °C, indicating the thermal decomposition of the Na alg. [16]. A broad endothermic peak was observed as a result of the dehydration process over a temperature range of 60–100 °C for HPMC K15M [17]. The DSC thermograph of the physical mixture consisting of BH, Na alginate, and HPMC K15M showed a broad endothermic peak at 75.61 °C and broad exothermic peak at 261.07 °C. Furthermore, the physical mixture did not retain the drug endothermic peaks, suggesting that the drug lost its crystalline properties and converted to an amorphous state. A DSC thermogram of the physical mixture showed no appearance of new peaks, indicating the compatibility between the drug and the polymers [17]. 

### 2.2. Physicochemical Evaluation of In Situ Gelling Solutions

At room temperature, all formulations were liquid and did not show any signs of gelation. It was found that the drug content percentage of the prepared formulations was between 85 and 99.52%, as shown in Table 1. These results indicate the homogenous drug distribution throughout the gelling solution. All the formulations had pH in the range of 6.9–7.7, which was found to be suitable for oral administration; therefore, there was no need to adjust the pH [18]. The in vitro gelling capacity of the in situ gelling formulations is demonstrated in Table 1. The viscosity of the formulations was taken into consideration during the selection of the in situ gelling system [19]. All the formulations remained liquid and suitable for oral administration. The formulations that contained 1% *w*/*v* of Na alg. with 0% and 0.3% HPMC and 1.5% *w*/*v* of Na alginate with 0% HPMC showed immediate gelation upon contact with the 0.1 N HCl and remained for 12 h (++) as they began to dissolve and erode, probably because of the weak cross-linking that resulted from the low polymer concentration [18]. All the other formulations showed immediate gelation and remained intact for more than 24 h (+++). As shown in Table 1, the formulations showed an increase in viscosity with increasing concentrations of both Na alginate and HPMC [13]. This can be attributed to the greater likelihood of chain interactions in the presence of high concentrations of the polymer [19,20]. 

### 2.3. In Vitro Release of BH from In Situ Forming Gels

**Figure 2:**
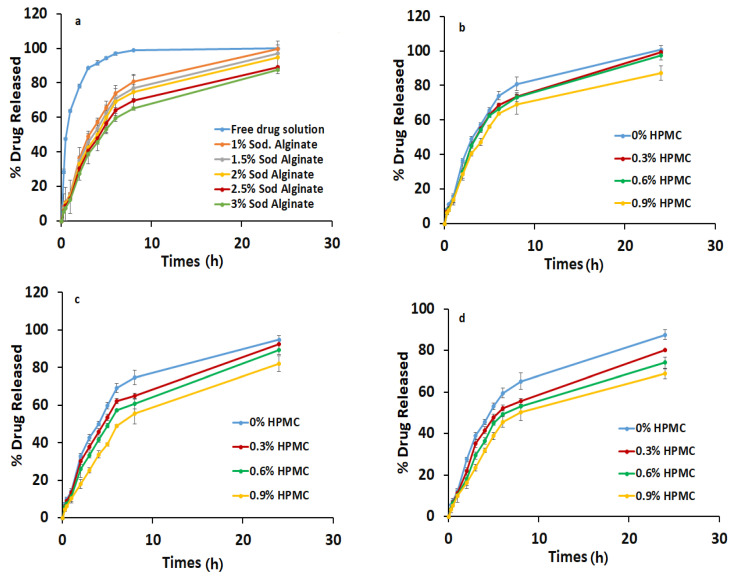
(**a**) Effect of Na alginate concentration on the in vitro release of BH from in situ gelling formulations. Effect of HPMC concentration on the in vitro release of BH from in situ gelling formulations containing (**b**) 1% Na alginate, (**c**) 2% Na alginate, and (**d**) 3% Na alginate.

According to the data of the in vitro release of BH from the different Na alg. in situ gels, the in vitro release rate of BH was slower than that detected for the free BH solution. Therefore, it was observed that the in situ gelling preparations had a high efficiency in decreasing the drug release rate compared with the free BH solution, which released about 95% within six hours, as shown in Figure 2.

#### 2.3.1. Effect of Sodium Alginate Concentration on the In Vitro Release of BH from In Situ Gelling Formulations

The effect of the Na alg. concentration on the in vitro release of the drug from the in situ gels was illustrated in Figure 2a. A significant decrease (*p* < 0.05) in the drug release was observed with the increase in Na alg. concentration; this was related to the increased aggregation of the polymer molecules and the increase in the diffusional path length, which the drug molecules ought to traverse [18]. Moreover, as the viscosity increased with increasing concentrations of the polymer, the solvent’s penetration into the core of the matrix was decreased, and the release of the drug was hindered [18].

#### 2.3.2. Effect of HPMC Concentration on the In Vitro Release of BH from In Situ Gelling Formulations

From Figure 2b–d, it can be observed that the HPMC had a release-retarding effect on all the chosen sodium alginate concentrations (1, 2, and 3% *w*/*v*). This could be explained by the fact that with an increase in HPMC concentration, the number of polymer particles is increased, thereby increasing the viscosity and retarding the release [21].

Moreover, HPMC forms a gel layer that prevents the diffusion of the dissolved calcium chloride out of the matrix quickly. Therefore, there is enough time for calcium ions to attach to the swelled alginate chain, forming a viscous gel within the matrix [22].

### 2.4. Data Analysis

#### 2.4.1. Full Factorial Experimental Design

A (3^3^) full factorial experimental design with three independent variables at three different levels was used to investigate the effect of three factors, sodium alginate concentration (X_1_), HPMC concentration (X_2_), and CaCl_2_ concentration (X_3_), on the viscosity (Y_1_) and percentage drug release from the in situ forming gel after 6 h (Y_2_). The transformed values of all the formulations, along with their results, are shown in Table 2. The viscosity (Y_1_) (dependent variable) values ranged from a minimum of 38.33 ± 1.53 cP to a maximum of 1660.0 ± 10.00), and the polynomial equation (full model) that described the response was:

(1) $$\begin{array}{c} Y_{1}(\mathrm{viscosity})\;=\;192.67\;+\;314.06X_{1}\;+\;40.204X_{2}\;+\;54.926X_{3}\;+\;116.03X_{1}X_{2}\;+\;152.72X_{1}X_{3}\;+\;75.22X_{2}X_{3}\;+\;\\ 187{X_{1}}^{2}\;+\;36.33{X_{2}}^{2}\;+\;62.33{X_{3}}^{2}\;R^{2}\;=\;0.9933\;(\mathrm{good}\;\mathrm{fit}) \end{array}$$

**Figure 3:**
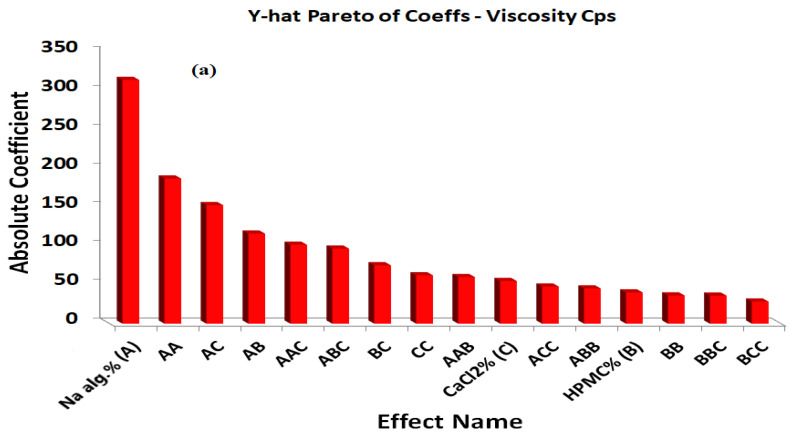

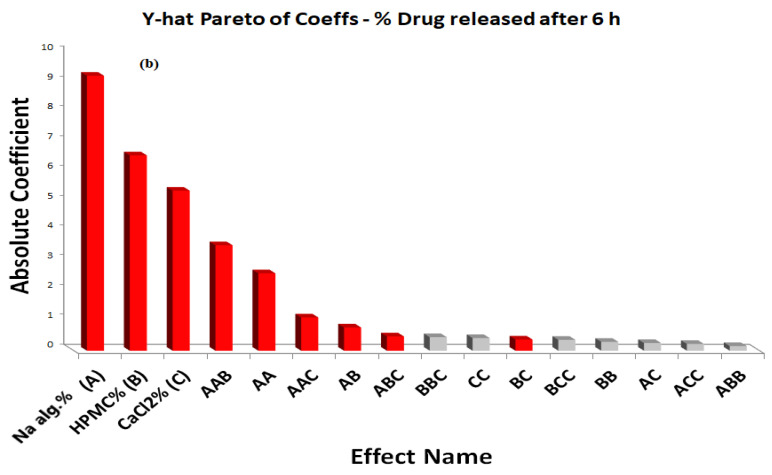
Y-hat Pareto chart showing (**a**) the standardized effect of independent variables and their interaction on the viscosity of the in situ forming gels, (**b**) the standardized effect of independent variables and their interaction on the percentage of drug released after 6 h from the in situ forming gels; (red color indicates significant effect; grey color indicates insignificant effect).

The positive coefficients of X_1_, X_2_, and X_3_ indicate that the viscosity increased with increases in the X_1_, X_2_, and X_3_ concentrations. The statistical analysis of the full model shows that the independent variables had a significant effect on the responses (*p* < 0.05). The standardized effect of the independent variables and the effect of their interaction on the viscosity were easily described by preparing a Pareto chart (Figure 3a). The theoretical (predicted) values and observed values were in reasonably good agreement, as shown in Table 2.

The percentage of the drug released after 6 h was found to be in the range of 35.24 to 68.59%. A polynomial equation was also developed for the percentage of the drug released after 6 h:

(2) $$\begin{array}{c} Y_{2}(\%\;\mathrm{drug}\;\mathrm{released}\;\mathrm{after}\;6\;h)\;=\;51.7\;-\;9.215X_{1}\;-\;6.545X_{2}\;-\;5.36X_{3}\;-\;0.78X_{1}X_{2}\;-\;0.26X_{1}X_{3}\;-\;0.37X_{2}X_{3}\;+\;\\ 2.595{X_{1}}^{2}\;-\;0.297{X_{2}}^{2}\;-\;0.423{X_{3}}^{2}\;R^{2}\;=\;0.9886\;(\mathrm{good}\;\mathrm{fit}) \end{array}$$

The negative coefficients for X_1_, X_2_, and X_3_ and the interactions between the two variables, X_1_X_2_, X_1_X_3_, X_2_X_3_, X_2_^2^, and X_3_^2^ indicated an unfavorable effect on the percentage of the drug released after 6 h, while the positive coefficient for X_1_^2^ indicates a favorable effect on the percentage of the drug released after 6 h. 

The statistical analysis of the full model shows that among the independent variables selected and their interactions, X_1_, X_2_, X_3_, X_1_X_2_, X_2_X_3_, X_1_^2^ were found to be significant (*p* < 0.05), indicating their major contribution to the percentage of the drug released after 6 h. The significance level of the coefficients b_5_, b_8_, and b_9_ was found to be more than 0.05 (*p* > 0.05); hence, they were omitted from the full model to generate the reduced model second-order polynomial Equation (3):

Y_2_ = 51.7 − 9.215X_1_ − 6.545X_2_ − 5.36X_3_ − 0.78X_1_X_2_ − 0.37X_2_X_3_ + 2.595X_1_^2^ (3)

The main effects of the independent variables and their interaction on the percentage of the drug released after 6 h were illustrated in a Pareto chart (Figure 3b). The theoretical (predicted) values and observed values were also in reasonably good agreement, as shown in Table 2.

From the analysis of variance (ANOVA) shown in Table S1, we can conclude that the model is highly significant. The F values of 0.000 for Y_1_ and 0.000 for Y_2_ indicated a significant effect of the independent factors on the responses Y_1_ and Y_2_. This implies that the main effect of the sodium alginate concentration percentage, the HPMC concentration percentage, and the CaCl_2_ concentration percentage is significant. Moreover, the non-significant lack-of-fit values for the two responses (*p* > 0.05), 30.6491 and 0.8361, and the consistent *p*-values of 0.1375 and 0.5964 for Y_1_ and Y_3_, respectively, indicated a good relation between the experimental and predicted values.

**Figure 4:**
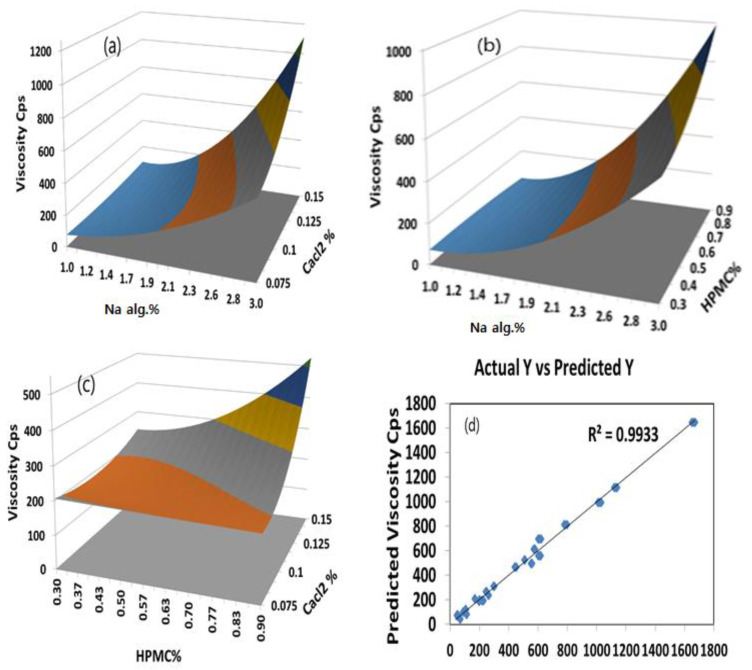
(**a**) Y-hat surface plot showing the effect of X_1_ and X_3_ on Y_1_ at constant X_2_ = 0; (**b**) Y-hat surface plot showing the effect of X_1_ and X_2_ on Y_1_ at constant X_3_ = 0; (**c**) Y-hat surface plot showing the effect of X_2_ and X_3_ on Y_1_ at constant X_1_ = 0; (**d**) linear correlation plot showing predicted against actual values to demonstrate the influence of independent variables X_1_, X_2_, and X_3_ on viscosity cP.

The surface plots were constructed for further clarification of the relationship between the dependent and independent variables. The effects of the interaction of each pair of factors on the viscosity at a fixed level of the third one (medium level) are shown in Figure 4. It was determined from the surface plots that a lower viscosity could be obtained with an X_1_ range from 1.0 to 2.4%, with all the X_2_ and X_3_ ranges at medium X_3_ and X_2_ levels, respectively, with a viscosity ranging from 10 to 310 cP. It is evident that increasing the level of X_1_ was the main cause of the increasing the viscosity. It was obvious that the sodium alginate concentration contributed more than the HPMC and CaCl_2_ concentrations to controlling the viscosity of the formulation. The percentage contribution of the sodium alginate concentration was found to be 68.15% versus 4.57% for the HPMC concentration and 10.15% for CaCl_2_ concentration. 

**Figure 5:**
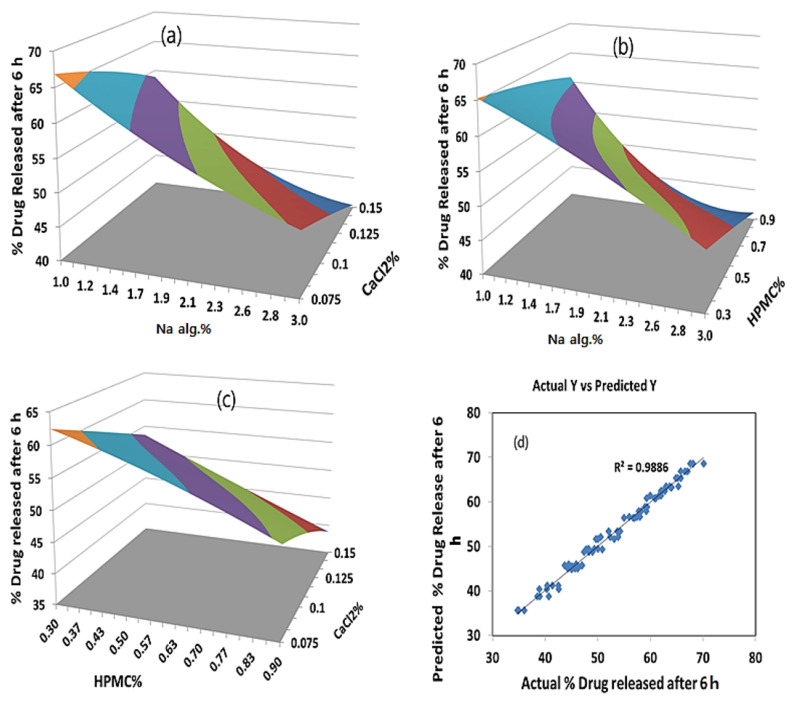
(**a**) Y-hat surface plot showing the effect of X_1_ and X_3_ on Y_2_ at medium level of X_2_; (**b**) Y-hat surface plot showing the effect of X_1_ and X_2_ on Y_2_ at medium level of X_3_; (**c**) Y-hat surface plot showing the effect of X_2_ and X_3_ on Y_2_ at medium level of X_1_; (**d**) linear correlation plot showing predicted against actual values to demonstrate the influence of independent variables X_1_, X_2_, and X_3_ on percentage of rug released after 6 h.

As far as the percentage of the drug released after 6 h is concerned, the effects of the interaction of each pair of factors on the percentage of the drug released after 6 h at a fixed level of the third one (medium level) are shown in Figure 5. It was determined from the surface plots that a lower value of Y_2_ could be obtained with an X_1_ level ranging from 1.8 to 3% in combination with an X_2_ level ranging from 0.36 to 0.9%, at a medium level of X_3_ and in combination with an X_3_ level in the range of 0.09 to 0.15%, and at a medium level of X_2_ with a percentage of drug released after 6 h ranging from 40% to 48%. It was obvious that the sodium alginate concentration contributed more than the HPMC and CaCl_2_ concentrations to controlling the percentage of the drug released after 6 h from the formulation. The percentage contribution of the sodium alginate concentration was found to be 65.43% versus 15.61% for the HPMC concentration and 14.82% for the CaCl_2_ concentration.

#### 2.4.2. The Optimum Formulation

The optimum formulation is the formulation that gives the minimum viscosity and a controlled drug release. It is evident from the polynomial equations and surface plots in Figure 4 and Figure 5 that increasing the sodium alginate concentration increased the viscosity and decreased the percentage of the drug released after 6 h. Hence, the medium level was selected as the optimum for the sodium alginate concentration percentage (X_1_). Using a computer optimization process, a 2% sodium alginate concentration (X_1_), a 0.9% HPMC concentration (X_2_), and a 0.1125 CaCl_2_ concentration (X_3_) were selected as the optimum formulation, which produced theoretical values of 269.2 cP and 44.9% for the viscosity and percentage of the drug released after 6 h, respectively.

### 2.5. Bioavailability of Orally Administered BH

**Figure 6:**
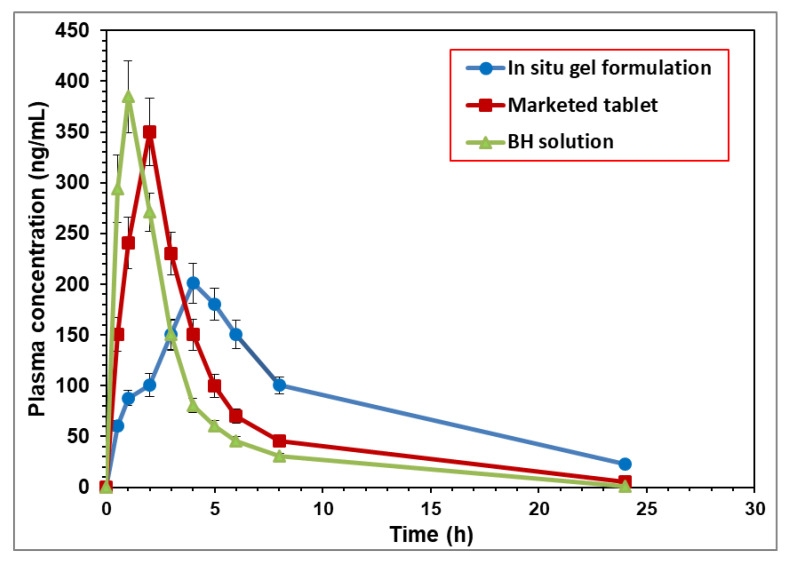
Mean plasma concentrations of BH after oral administration of different formulations (equivalent to 10 mg/kg) to rabbits.

The mean plasma concentrations of BH per duration of the three preparations, the BH solution, the BH marketed tablet, and the optimized BH in situ gel formulation, are illustrated in Figure 6. From the obtained results, it was evident that there was a difference between the mean plasma concentrations of the in situ gel formulation at all the time intervals compared to the plain drug and the marketed tablet. Furthermore, there was a marked difference in the T_max_ between the plain drug and the tested formulation. 

The mean pharmacokinetic parameters of the BH from the different formulations are summarized in Table S2 and represented by the value of C_max_ (ng/mL), T_max_ (h), K_el_ (h^−1^), t_1/2_ (h), AUC_0–24_ (ng·h·mL^−1^), AUC_0-∞_ (ng·h·mL^−1^), AUMC_0-∞_ (ng·h^2^·mL^−1^), and MRT (h). From the observed data, it was noticed that the absorption of the BH from the BH solution was fast and reached its peak plasma concentration in 0.75 ± 0.04 h, whereas the mean T_max_ for the marketed tablet and the tested formulation were 2.00 ± 0.27 h and 4.00 ± 0.30 h, respectively. The mean peak plasma concentrations (C_max_) were 350.36 ± 33.22 ng/mL for the marketed product and 200.95 ± 19.43 ng/mL for the in situ gel formulation compared to 384.82 ± 35.48 ng/mL for the BH solution. The obtained results showed a decrease in the mean C_max_ and an increase in the mean T_max_ of the drug-loaded in situ gel formulation compared to the plain drug solution, which explained the controlled release of the BH in situ gel formulation. The mean AUC_0–24_ was found to be 2041.19 ± 200.16 ng·h·mL^−1^ for the in situ gel formulation compared to 1350.87 ± 136.05 ng·h·mL^−1^ for the BH solution. The mean residence time for the BH when released from the in situ gel was significantly longer than that following the oral administration of the drug in the solution [23]. As seen in Table S2, the in situ gel formulation increased the MRT of the BH by 3.33-fold compared to free drug solution and resulted in an improved bioavailability. 

From the obtained data, it was concluded that the relative bioavailability of the BH-loaded in situ gel was higher than the drug solution by more than 1.5-fold. The enhanced relative bioavailability of the BH may have been due to the decreased release of the drug from the in situ gel, which led to a more sustained release and absorption of the drug compared to the free-solution form [15,24].

## 3. Conclusions

A novel oral in situ gel system for the sustained delivery of BH was developed utilizing polymers that exhibit solution-to-gel phase transition due to changes in pH. In situ gel formation depends on the presence of sodium citrate, which complexes with free Ca ions to maintain the in situ gel fluidity until it reaches the stomach. The in situ gel formulation viscosity showed a marked increase with increases in the Na alginate concentration, with a significant decrease in the rate and extent of drug release, as proven by the (3^3^) full factorial design. The derived polynomial equations, contour, and surface plots helped to predict the values of the selected independent variables for the preparation of optimum in situ gelling formulations with the desired properties. The formulation containing 2% Na alginate concentration (X_1_), 0.9% HPMC concentration (X_2_), and 0.11 CaCl_2_ concentration (X_3_) was selected as the optimum formulation, which showed a 1.5-fold increase in bioavailability in comparison to the drug solution.

## 4. Materials and Methods

### 4.1. Materials

Buspirone HCl (BH) and sodium alginate (Na alg.) were gift samples kindly supplied by Sigma Pharmaceuticals, Quesna, Egypt. Hydroxypropyl Methylcellulose (HPMC K15M) was supplied as a gift sample by the Egyptian International Pharmaceutical Industries Co., (EPICO), El-Asher of Ramadan city, Egypt. Sodium citrate, calcium chloride, and hydrochloric acid were purchased from El-Nasr Pharmaceutical Chemical Co., Cairo, Egypt.

### 4.2. Preparation of In Situ Gelling Solution

Different HPMC amounts to produce final concentrations of 0.3, 0.6, and 0.9% *w*/*v* were dissolved in around 50% of the total amount of distilled water containing calcium chloride (0.075, 0.1, 0.15% *w*/*v*), sodium citrate (0.25% *w*/*v*), and BH (1 mg/mL), so that there was a proper and homogenous dispersion of BH in the solution. Sodium alginate at different concentrations (1.0, 1.5, 2.0, 2.5, and 3.0% *w*/*v*) was added to the other half of distilled water and then heated to 60 °C while stirring. After cooling, these two solutions were thoroughly mixed using magnetic stirrer (AREC Digital Ceramic Hot Plate Stirrer, Usmate Velate (MB)-Italy) [25,26].

### 4.3. Differential Scanning Calorimetry Studies (DSC)

The DSC thermograms were recorded using a Differential scanning calorimeter (Model DSC-50, Shimadzu Corporation, Kyoto, Japan). About 2 mg of samples were sealed in aluminum pans and heated over a temperature range of 0–300 °C at a constant rate of 10 °C/min under a nitrogen purge (30 mL/min) [27,28]. DSC thermograms of pure BH, Na alg., and HPMC were taken to identify their characteristic endothermic peaks in order to determine any possible interactions in the physical mixtures of the drug and polymer. 

### 4.4. Determination of Drug Content

In total, 1 mL of the in situ gelling solution (equivalent to 1 mg of BH) was diluted in 100 mL of distilled water to yield a solution containing a theoretical strength of 10 μg/mL. The UV absorbance of the sample was determined at a wavelength of 239 nm using a blank containing the same components of the gelling solution, exempt the drug [29]. The test was repeated 3 times, and percentage drug content was detected according to the following equation [30,31]:

(4) $$\%\;\mathrm{Drug}\;\mathrm{content}=\frac{\mathrm{Actual}\;\mathrm{amount}\;\mathrm{of}\;\mathrm{the}\;\mathrm{drug}\;\mathrm{in}\;\mathrm{the}\;\mathrm{formulation}}{\mathrm{Theoretical}\;\mathrm{amount}\;\mathrm{of}\;\mathrm{the}\;\mathrm{drug}\;\mathrm{in}\;\mathrm{the}\;\mathrm{formulation}}\times 100$$

### 4.5. Measurement of pH

The pH measurement of sodium-alginate-based in situ gelling solutions were performed using a calibrated digital pH meter (Cole-parmer instrument Co., Vernon Hills, IL, USA) at room temperature [32,33].

### 4.6. Gelling Capacity

In vitro gelling capacity was assessed visually by transferring 5 mL of each formulation into 25 mL of the gelation solution (0.1 N HCL, pH 1.2) in a beaker and observing the gelation time and how long the formed gel remained intact. Formulations were graded as follows [18,34]:

(+) Gels after a few minutes, dispersed rapidly.

(++) Gelation is immediate and remains for 12 h.

(+++) Gelation is immediate and remains for more than 12 h.

### 4.7. Measurement of Viscosity

The viscosity of the prepared in situ gelling solutions was determined by viscostar-R rotational viscometer (Fungilab S.A., Barcelona, Spain) using 100-milliliter sample. The study was carried out at 25 °C and 100 rpm using suitable spindle number R2 [26].

### 4.8. In Vitro Drug Release Study from In Situ Gels

The 10-milliliter in situ gelling solution containing 10 mg BH was poured into a glass tube with a cellophane membrane (mol.wt cutoff = 10.000 D) on one side and suspended in a beaker containing 100 mL of 0.1 N HCL, pH 1.2 to maintain sink condition. The beaker was placed in a mechanical shaker water bath (Julabo Shaking water bath SW–20C, Berlin, Germany) and agitated at 100 rpm while maintaining a temperature of 37 ± 1 °C [11]. Two-milliliter samples were taken at different time intervals for 24 h and the amount of drug was spectrophotometrically determined at 239 nm using buffer pH 1.2 as a blank. Each sample was replaced with an equal volume of fresh buffer solution, pH 1.2, at 37 ± 0.5 °C [13]. First, the effect of different concentrations of Na alg. on the drug release was investigated; next, a preliminary investigation of the influence of changing HPMC concentration on three concentrations of Na alg. (1%, 2% and 3%) was also performed. 

### 4.9. Data Analysis

#### 4.9.1. Factorial Experimental Design

The approach of changing one variable at a time is traditionally used for the development of pharmaceutical formulations, but there are drawbacks to this approach, such as its high consumption of time and raw materials. Furthermore, it may be difficult to predict the combined effects of various independent variables on the end product of the formulation process. This is why it is essential to comprehend the intricacy of pharmaceutical formulations by using a factorial-design statistical program, which is an effective method of demonstrating the relative significance of various variables and their associations [35].

Quantum XL^®^ Version 5.50 software (SigmaZone, Orlando, FL, USA, accessed on 4 June 2021). was used to conduct the study. The present study used a three-level three-factorial (3^3^) design for experimentation with 3 factors, 3 levels, and 27 runs to study the effect of independent variables, concentration of sodium alginate (X_1_), concentration of CaCl_2_ (X_2_), and concentration of HPMC (X_3_), on the dependent variables’ viscosity (Y_1_) and percentage drug release after 6 h (Y_2_).

The independent variables and their levels were listed in Table 3. The concentrations of X_1_, X_2_, and X_3_ were selected based on the results of preliminary experiments. The following quadratic mathematical model equation is used to clarify the effects of independent variables on the responses:

Y = b_0_ + b_1_X_1_ + b_2_X_2_ + b_3_X_3_ + b_4_X_1_X_2_ + b_5_X_1_X_3_ + b_6_X_2_X_3_ + b_7_X_1_^2^ + b_8_X_2_^2^ + b_9_X_3_^2^ (5)

where Y is the dependent variable, b_0_ is the intercept, b_1_ to b_9_ are the regression coefficients measured from the observed experimental values of Y during the experimental runs, and X_1_, X_2_, and X_3_ represent the average results of changing one variable at a time from its lowest level to its highest level. X_1_X_2_, X_1_X_3_, and X_2_X_3_ show how the dependent variable changes when two variables are changed. The terms (b_4_, b_5_, b_6_) and (b_7_, b_8_, b_9_) represent the interaction and quadratic terms, respectively [36,37]. Optimization was performed to determine the levels of the independent variables (X_1_, X_2_, and X_3_) that would produce a minimum value of viscosity and extended drug release.

#### 4.9.2. Statistics

All results were expressed as mean ± SD. Three runs were used to calculate the mean value. ANOVA test was used for comparison of sample means and for determination of statistical significance. Statistically, all the results were considered significant if *p* < 0.05.

### 4.10. Bioavailability Study of BH after Oral Administration to Experimental Animals

White male albino rabbits (weighing 2–2.5 kg) were selected for the bioavailability studies. All animal approaches were in accordance with the accepted protocol for experimental animals organized by the Research Ethics Committee (REC) of Ha’il University (20455/5/42). All animals were fasted for 12 h before the experiments with free water access. This examination was designed as a single oral dose. All animals received 10 mg BH/kg of body weight [38]. Animals were divided into three groups of three rabbits each, as follows:

Group 1 received BH solution in distilled water.

Group 2 received BH marketed product (Buspar ^®^ tablet).

Group 3 received the optimized oral BH in situ gel.

About 2.5 mL of blood samples were withdrawn from the sinus orbital into heparinized tubes at different time durations: 0.5, 1, 2, 3, 4, 5, 6, 8, and 24 h. The blood samples were centrifuged immediately using centrifuge (Hermle Labortechnik GmbH-vZ 300 K, Wehingen, Germany) at 4000 rpm for 10 min at 4 °C to obtain the plasma samples, which were then stored at −20 ± 0.5 °C until HPLC analysis.

For analysis, BH content was assessed using HPLC with a variable -wavelength PDA detector, according to the technique described by Bshara et al. [39], with slight modifications. Aliquot of 500 µL of each thawed plasma sample was mixed with one mL of acetonitrile. The mixture was vortex-mixed for 30 s and then centrifuged at 6000 rpm for 15 min. The HPLC system (ThermoScientific Surveyor Plus HPLC system, ThermoScientific company, Waltham, MA, USA) consisting of Hypersil gold C18 column (particle size 5 µm, 150 × 4.6 mm) was conditioned at 30 °C and eluted with a mobile phase consisting of acetonitrile and a potassium phosphate buffer (10 mM) 30:70 (*v*/*v*) adjusted to pH 4.6 with orthophosphoric acid at a flow rate of 0.7 mL/min and an injection volume of 25 µL. The effluent was monitored at 235 nm.

Pharmacokinetic parameters (C_max_, T_max_, AUC_0–24_, AUC_0–∞_, AUMC_0–∞,_ t_1/2_, and mean residence time (MRT)) in plasma were calculated using the noncompartmental model in the WinNonlin Standard Edition Version1.1 program (Pharsight, Mountain View, CA, USA) [40]. Bioavailability of BH nanovesicular in situ gel formulation relative to the BH solution was calculated using the following equation:

(6) $$\mathrm{Relative}\;\mathrm{bioavailability}=\frac{\mathrm{AUC}\;0-24\;\left(\mathrm{tested}\;\mathrm{formulation}\right)}{\mathrm{AUC}\;0-24\;\left(\mathrm{control}\right)}\times 100$$

## Figures and Tables

**Table 1:** Composition of the in situ gel formulations, their viscosity, pH, drug content, and gelling capacity.

Na Alginate Conc. % *w*/*v*	HPMC K-15M Conc. % (*w*/*v*)	Viscosity (cP)	pH	Drug Content % (*w*/*v*)	Gelling Capacity
1	0.0	30.00 ± 0.50	6.90	96.00 ± 0.20	++
	0.3	38.33 ± 1.53	7.25	98.96 ± 0.37	++
	0.6	50.33 ± 3.06	7.30	97.20 ± 0.55	+++
	0.9	52.33 ± 0.58	7.41	95.89 ± 0.63	+++
1.5	0.0	49.00 ± 3.60	7.32	99.00 ± 1.15	++
	0.3	103.6 ± 3.00	7.40	99.27 ± 0.24	+++
	0.6	125.0 ± 2.55	7.46	98.85 ± 0.62	+++
	0.9	139.6 ± 1.40	7.50	89.60 ± 0.46	+++
2	0.0	100.5 ± 0.44	7.44	98.00 ± 0.88	+++
	0.3	169.0 ± 1.00	7.53	96.40 ± 0.38	+++
	0.6	199.7 ± 1.53	7.55	99.52 ± 0.42	+++
	0.9	227.0 ± 1.00	7.62	92.40 ± 0.49	+++
2.5	0.0	177.0 ± 2.00	7.70	98.00 ± 1.36	+++
	0.3	308.0 ± 2.32	7.73	95.20 ± 0.57	+++
	0.6	377.0 ± 2.88	7.75	92.70 ± 0.90	+++
	0.9	401.4 ± 3.00	7.77	90.50 ± 0.53	+++
3	0.0	300.0 ± 4.00	7.70	97.00 ± 0.36	+++
	0.3	447.0 ± 6.08	7.76	85.00 ± 0.24	+++
	0.6	555.0 ± 5.00	7.77	87.00 ± 0.78	+++
	0.9	575.7 ± 5.13	7.78	88.42 ± 0.73	+++

All formulations contained 0.25% sodium citrate and 0.075% calcium chloride. (++) formulations showed immediate gelation and remained for 12 h; (+++) formulations showed immediate gelation and remained intact for more than 24 h. Each result is the mean of three determinations ± standard deviation (SD).

**Table 2:** Observed responses in (3^3^) factorial experimental design for BH in situ forming gel formulations.

Formulation No.	Independent Variables			Dependent Variables			
	X_1_	X_2_	X_3_	Observed Value of Y_1_	Predicted Value of Y_1_	Observed Value of Y_2_	Predicted Value of Y_2_
1	3	0.9	0.15	1660 ± 135	1647.7	35.25 ± 0.68	35.69
2	3	0.9	0.11	1020 ± 98	991.73	41.41 ± 1.07	41.15
3	3	0.9	0.075	575 ± 45	613.00	45.48 ± 0.74	45.49
4	3	0.6	0.15	1130 ± 103	1113.1	40.61 ± 1.87	40.38
5	3	0.6	0.11	610 ± 40	693.72	45.41 ± 0.97	45.08
6	3	0.6	0.075	555 ± 37	494.63	49.19 ± 0.92	49.39
7	3	0.3	0.15	787 ± 30	814.33	45.88 ± 1.89	45.71
8	3	0.3	0.11	609 ± 29	559.21	48.20 ± 0.80	48.73
9	3	0.3	0.075	447 ± 16	467.54	52.26 ± 1.67	52.09
10	2	0.9	0.15	509 ± 21	526.24	39.38 ± 1.17	38.79
11	2	0.9	0.11	246 ± 12	269.20	45.00 ± 0.03	44.86
12	2	0.9	0.075	227 ± 12	193.72	48.98 ± 1.65	49.35
13	2	0.6	0.15	299 ± 12	309.93	45.40 ± 0.83	45.92
14	2	0.6	0.11	217 ± 10	192.67	50.95 ± 1.92	51.70
15	2	0.6	0.075	199 ± 13	200.07	57.17 ± 1.05	56.64
16	2	0.3	0.15	261 ± 11	238.50	53.35 ± 1.11	53.36
17	2	0.3	0.11	197 ± 13	188.79	58.42 ± 0.75	57.95
18	2	0.3	0.075	169 ± 13	206.87	62.33 ± 0.41	62.43
19	1	0.9	0.15	90 ± 1.00	102.07	56.24 ± 1.10	56.38
20	1	0.9	0.11	69 ± 1.00	40.73	60.38 ± 0.94	60.82
21	1	0.9	0.075	52 ± 0.58	65.28	63.63 ± 0.59	63.24
22	1	0.6	0.15	112 ± 11	83.93	59.12 ± 0.21	58.87
23	1	0.6	0.11	57 ± 1.00	65.61	63.99 ± 1.22	63.51
24	1	0.6	0.075	50 ± 3.00	76.29	66.48 ± 0.64	66.85
25	1	0.3	0.15	106 ± 11	119.83	61.21 ± 1.09	61.36
26	1	0.3	0.11	47 ± 0.58	72.32	65.31 ± 0.46	65.29
27	1	0.3	0.075	38 ± 2.00	38.07	68.59 ± 1.36	68.64

X_1_: Na alg. concentration; X_2_: HPMC concentration; X_3_: CaCl_2_ concentration; Y_1_: viscosity cP; Y_2_: percentage of drug released after 6 h.

**Table 3:** Variables and their levels in the 3^3^ factorial design.

Independent Variables	Levels		
	Low (−1)	Medium (0)	High (1)
X_1_ = Sodium alginate concentration (%). X_2_ = Cacl_2_ concentration (%). X_3_ = HPMC concentration (%).	1 0.075 0.3	2 0.1125 0.6	3 0.15 0.9
Dependent variables	Constraints		
Y_1_ = Viscosity. Y_2_ = % Drug released after 6 h.	Minimize Prolong		

## Data Availability

Not applicable.

## Supplementary Materials

The following supporting information can be downloaded at: https://www.mdpi.com/article/10.3390/gels8070395/s1. Table S1: Results of ANOVA test for viscosity and percentage of drug released after 6 h of in situ forming gels. Table S2: Pharmacokinetics parameters after oral administration of BH in various formulations.
