# Supplementary material for: Response Surface Methodology for Optimization of Buspirone Hydrochloride-Loaded In Situ Gel for Pediatric Anxiety
Source: Gels. 2022 Jun 22;8(7):395. doi: 10.3390/gels8070395 (PMC9323393; doi:10.3390/gels8070395)
Supplement: Supplementary file 1 [file gels-08-00395-s001.zip › gels-1733803-supplementary.pdf]

gels

MDPI

Article

Response Surface Methodology for Optimization of Buspirone Hydrochloride-Loaded In Situ Gel for Pediatric Anxiety

Marwa H. Abdallah [1,2,*], Dina M. Abdelnabi [2] and Hanaa A. Elghamry [2]

[1] Department of Pharmaceutics, College of Pharmacy, University of Ha'il, Hail 81442, Saudi Arabia.
[2] Department of Pharmaceutics and Industrial Pharmacy, Faculty of Pharmacy, Zagazig University, 44519 Zagazig, Egypt; Dinaabdelnabi@zu.edu.eg (D.M.A.); haelghamry@zu.edu.eg (H.A.E.)
* Correspondence: mh.abdallah@uoh.edu.sa

Supplementary Materials

Table S1. Results of ANOVA test for viscosity and % drug released after 6 h of *in-situ* forming gels.

Regression	Df	SS	MS	F value	Sig F (P value)	FLOF	Sig FLOF
Viscosity	16	12,057855	75,3616	590	0.0000	30.6491	0.1375
% drug released after 6 h	16	6731	420.7	346.3	0.0000	0.8361	0.5964

ANOVA indicates analysis of variance; Df, degrees of freedom; SS, sum of squares; MS, mean of squares; F, Fischer's ratio, P < 0.05 level.

Table S2. Pharmacokinetics parameters after oral administration of BH in various formulations.

Parameters	Formulation		
	BH solution	Marketed tablet	*In-situ* gel Formulation
C_{max} (ng/ml)	384.82 ± 35.48	350.36* ± 33.22	200.95* ± 19.43
T_{max} (h)	1.00 ± 0.04	2.00* ± 0.27	4.00* ± 0.30
K_{el} (h^{-1})	0.20 ± 0.02	0.18* ± 0.01	0.10* ± 0.00
$t_{1/2}$ (h)	3.55 ± 0.34	3.86* ± 0.39	6.53* ± 0.59
AUC_{0-24} ($ng.ml^{-1}.h$)	1350.87 ± 136.05	1646.30* ± 155.77	2041.19* ± 200.16
$AUC_{0-\infty}$ ($ng.ml^{-1}.h$)	1357.84 ± 132.69	1682.03* ± 164.22	2258.13* ± 212.19
$AUMC_{0-\infty}$ ($ng.ml^{-1}.h^2$)	5042.51 ± 488.77	8738.63* ± 845.54	22686.76* ± 2266.77
MRT (h)	3.71 ± 0.29	5.20* ± 0.54	10.05* ± 0.99
Relative bioavailability	-------	--------	151.10

(*) the mean difference is significant at P<0.05 level. Each result is the mean of 3 determinations ± S.D.
